# Renal Tubular Acidosis in Pregnant Critically Ill COVID-19 Patients: A Secondary Analysis of a Prospective Cohort

**DOI:** 10.3390/jcm11154273

**Published:** 2022-07-22

**Authors:** Simona Humbel, Pedro David Wendel-Garcia, Simone Unseld, Fabienne Noll, Reto Andreas Schuepbach, Christoph Camille Ganter, Harald Seeger, Sascha David, Rea Andermatt

**Affiliations:** 1 Institute of Intensive Care Medicine, University Hospital Zurich, 8091 Zurich, Switzerland; simona.humbel@usz.ch (S.H.); pedrodavid.wendelgarcia@usz.ch (P.D.W.-G.); simone.unseld@usz.ch (S.U.); reto.schuepbach@usz.ch (R.A.S.); christoph.ganter@usz.ch (C.C.G.); rea.andermatt@usz.ch (R.A.); 2 Department of Obstetrics, University Hospital Zurich, 8091 Zurich, Switzerland; fabienne.noll@usz.ch; 3 Division of Nephrology, University Hospital Zurich, 8091 Zurich, Switzerland; harald.seeger@usz.ch

**Keywords:** renal, kidney, tubular, pregnancy, acidosis, intensive care unit, acid–base

## Abstract

Background: Renal tubular acidosis (RTA) is an extremely rare cause of metabolic acidosis (10 in 100,000). RTA has been linked neither to pregnancy nor to severe coronavirus disease 2019 (COVID-19). The purpose of this study was to analyze the prevalence and clinical course of normal anion gap metabolic acidosis in critically ill pregnant COVID-19 patients and to compare them to an age-matched nonpregnant female patient cohort. Methods: Secondary analysis was conducted on a prospective observational cohort of critically ill patients suffering from COVID-19 consecutively admitted to a tertiary intensive care unit (ICU) between February 2020 and April 2021. Results: A total of 321 COVID-19 patients required admission to the ICU; 95 (30%) were female, and 18 (19%) were of childbearing age. Seven of eight (88%) pregnant women (all in the last trimester) required advanced respiratory support due to COVID-19. The estimated glomerular filtration rate was 135 (123–158) mL/min/m^2^ body surface area, and six pregnant women (86%) were diagnosed with a normal, respiratory compensated, anion gap metabolic acidosis (pH_min_ 7.3 (7.18–7.31), HCO_3_^−^_min_ 14.8 (12.8–18.6) mmol/L, and paCO_2_ 3.4 (3.3–4.5) kPa). Three (43%) acidotic pregnant women fulfilled diagnostic criteria for RTA. All women recovered spontaneously within less 7 days. Conclusions: Metabolic acidosis seems to be very common (85%) in pregnant critically ill COVID-19 patients, and the prevalence of RTA might be higher than normal. It remains to be demonstrated if this observation is an indirect epiphenomenon or due to a direct viral effect on the tubular epithelium.

## 1. Introduction

Research on the novel coronavirus disease (COVID)-19 induced by the severe acute respiratory syndrome coronavirus 2 (SARS-CoV-2) pandemic has extensively focused on respiratory and multiple organ failure [1]. Considering the ability of the virus to infect the endothelium [2] in most vascular beds via its entry receptor angiotensin-converting enzyme 2 (ACE2) receptor, the virus has also been detected in renal endothelial and tubular epithelial cells [3], a high prevalence of acute kidney injury (AKI) has been described. However, relatively little is known about specific effects of the virus on tubular transport mechanisms.

Metabolic acidosis is generally no stranger to the intensivist and is most often a consequence of AKI or lactic acidosis [4,5]. If glomerular filtration rate (GFR) and anion gap (AG) are normal, the diagnostic approach involves urine analysis, and the differential diagnoses are more complex, involving very rare disorders such as renal tubular acidosis (RTA) (10 in 100,000) [6]. The hypokalemic RTAs are divided into the classical distal (type 1) and the proximal type (type 2). Whereas the proximal type’s primary mechanism is a reduced capacity to reabsorb filtered bicarbonate (HCO_3_^−^), distal RTA is characterized by defective excretion of hydrogen ions (H^+^) in the late distal convoluted tubule and in the collecting tubule and, therefore, an inability to adequately lower urine pH despite acidemia [7].

Characteristics of the distal RTA include typically a normal or near normal GFR, normal serum AG, and a urine pH > 5.3 with a urine AG > 20 meq/L. Plasma HCO_3_^−^ is variable but often lowered to 10–20 mmol/L, and plasma potassium is usually reduced. More common is the so-called incomplete distal RTA with normal plasma HCO_3_^−^ but inability to extensively lower urine pH [8].

During pregnancy, physiological adaptations lead to an increase in renal blood flow and, thus, GFR [9]. Serum creatinine levels, after declining in the beginning of pregnancy, increase to pre-pregnancy values in the third trimester [10]. Early in pregnancy, most likely due to changes in progesterone levels, mild respiratory alkalosis with a reduction in the partial arterial CO_2_ (paCO_2_) of up to 10 mmHg ensues, which usually lasts until term. Compensatory renal HCO_3_^−^ excretion is increased, leading to a mildly reduced HCO_3_^−^ around 18 to 22 mmol/L. Consequently, pregnant women are physiologically known to have a lower buffer capacity and are more susceptible to develop metabolic acidosis [11].

Women with known distal RTA have a higher risk of complications during pregnancy and a tendency toward worsening of the pre-existing RTA [12,13]. These patients are at risk for metabolic acidosis and severe hypokalemia, even leading to paralysis and rhabdomyolysis. It was emphasized that close nephrological monitoring of these patients is necessary [12]. Spontaneous development of RTAs during pregnancy has been reported but seems to be rather sparse [14].

We recently noticed that several pregnant critically ill COVID-19 patients presented with or developed normal-AG metabolic acidosis. Therefore, we systematically analyzed its prevalence and clinical course along with a comparison to an age-matched nonpregnant control group.

## 2. Materials and Methods

This study was planned as a secondary analysis of a prospectively collected COVID-19 cohort at the Institute of Intensive Care Medicine of the University Hospital Zurich, an academic tertiary care referral center, between February 2020 and April 2021. Both the prospective study protocol (ClinicalTrials.gov Identifier: NCT04410263, BASEC ID: ZH 2020-00646) and the secondary analysis protocol (BASEC ID: ZH 2021-01569) were approved by the regional cantonal ethical commission in Zurich. Informed consent was obtained either written or orally by the patients or from their next of kin. Written informed consent for publication from the patient or in case of death or disability, from the next of kin or legal representative, was sought for every patient included in the study. The study complies with the Declaration of Helsinki, the Guidelines on Good Clinical Practice issued by the European Medicines Agency, and Swiss law. All data analyzed and discussed in the framework of this study are included in this published article and its online Supplementary Materials. The corresponding author may provide specified analyses or fully deidentified parts of the dataset upon reasonable request.

### 2.1. Patient Population

**Figure 1:**
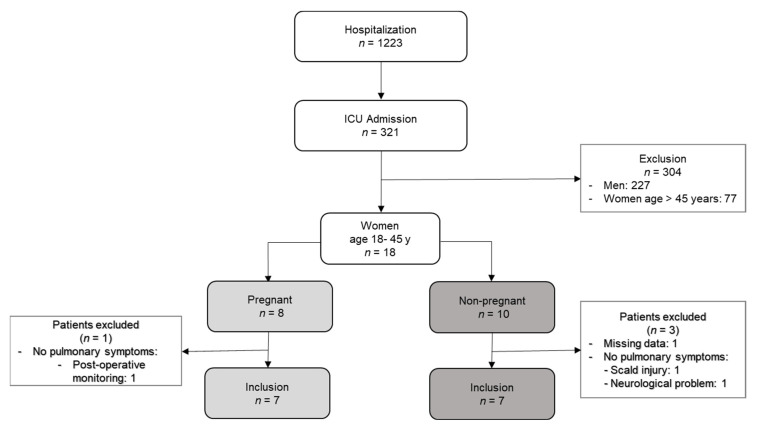
Flow chart of inclusion. Abbreviations: *n* (number).

All COVID-19 patients admitted to the ICU of our institute in the above-described 14 month period were included into the prospective observational cohort. For this secondary analysis, only women of childbearing age between 18 to 45 years were considered. Both pregnant women and those requiring emergency cesarean section (C-section) before admission to the ICU were included in the group of pregnant critically ill patients. Patients without requirement of respiratory support or a lack of documented acid–base characteristics were excluded. Figure 1 summarizes the group composition in a flow chart.

### 2.2. Study Design, Data Collection

Patients’ characteristics were subject to continuous acid–base monitoring by arterial blood gas analysis (ABG) sampling at least every 6 h. We additionally monitored urine samples, and we performed daily hematological and clinical chemical blood analyses. To determine the overall severity of the disease, the Sequential Organ Failure Assessment (SOFA) score was calculated daily, and the Simplified Acute Physiology Score II (SAPS) was calculated 24 h after ICU admission.

### 2.3. Statistical Analysis

Comparisons of population characteristics were performed using the Wilcoxon signed-rank and the chi-squared test, as appropriate. Statistical analysis was performed using SigmaPlot (SigmaPlot for Windows Version 14.0 Build 14.0.0.124, SPSS Inc., Chicago, IL, USA). A two-sided *p* < 0.05 was considered statistically significant. Values are given as medians with interquartile ranges (IQR) or counts and percentages as appropriate.

## 3. Results

### 3.1. Cohort Description/Demographics

During the observation period, 321 critically ill COVID-19 patients were admitted to the ICU of the University Hospital Zurich and, therefore, included in the study. Ninety-five (30%) were women and 18 were of childbearing age from 18 to 45 years. Eight of them were either pregnant or recently had a C-section before admission to the ICU (age 24 to 42 years). All of them were in the third trimester (31 (30–33) gestational weeks). One SARS-CoV-2-positive, but asymptomatic pregnant woman who was admitted to the ICU for post-surgery monitoring was excluded from the analysis. All seven symptomatic pregnant women suffered from fever and cough; five had dyspnea at hospitalization. None of the patients suffered from diarrhea. Three women were severely hypoxemic with an oxygen saturation below 90%. All pregnant women needed respiratory support, five pregnant women (71%) were treated with high-flow oxygen therapy (HFOT), and one of these women was later intubated. Three pregnant women needed respiratory support with invasive mechanical ventilation (MV) (43%).

Among the age-matched control group of the 10 nonpregnant COVID-19, two were excluded due to a lack of respiratory symptoms (hospitalization due to scalding and due to neurological problems). One woman was excluded due to missing acid–base data (Figure 1).

Three of the included pregnant women (43%) were suffering from gestational diabetes, and four (57%) were obese (BMI 35 (26–43) kg/m^2^), whereas, in the nonpregnant control group, only three patients (43%) were obese, and two had prediabetes. Outside of obesity and diabetes, 71% of the nonpregnant control group had other pre-existing comorbidities (e.g., heart failure, arterial hypertension, polycystic ovarian syndrome, anorexia, trisomy 21, and follicular lymphoma), whereas only one pregnant woman (14%) had another pre-existing condition (asthma and sarcoma).

Pregnant women stayed for 12 (10–20) days in the hospital and 5 (5–8) days in the ICU, whereas the nonpregnant women stayed for 28 (12–40) days in the hospital with an ICU stay of 15 (10–36) days.

Respiratory support (Table 1) was needed in 71% of the pregnant women provided by HFOT. Two women were intubated in the further course, and one underwent primary intubation. In the nonpregnant control group, five women were intubated, one after failure of HFOT at first, while two others had only HFOT. Three women also needed additional extracorporeal membrane oxygenation (ECMO). All pregnant patients survived the hospital stay, whereas there was one death in the nonpregnant control group.

Six pregnant (86%) and six nonpregnant (86%) women received an anti-inflammatory strategy with dexamethasone. In line with our in-house standard (where antiviral strategies are only given in the early course of the disease and mostly before ICU admission), treatment with remdesivir was only given to three nonpregnant patients. Two of the nonpregnant patients were additionally treated with tocilizumab and one patient received convalescent plasma.

SOFA scores at admission and the highest SOFA scores during the first week, as well as the SAPS scores, were not different between the two groups (Table 2).

### 3.2. Laboratory Parameters

Inflammation parameters such as C-reactive protein (CRP) and procalcitonin (PCT) were not different in the two groups, but ferritin was higher (Table 2) in the nonpregnant control group. Blood urea nitrogen (BUN) was lower in the pregnant women (1.1 (0.8–1.2) mmol/L vs. 3.1 (2.6–6.2) mmol/L; *p* = 0.02). According to eGFR (CKD-EPI 2009), there was no difference in renal function of the two groups. Serum potassium was equal in the pregnant (3.1 (2.8–3.7) mmol/L) and in the control group (3.9 (3.4–4.2) mmol/L).

At admission, the pH and HCO_3_^−^ of the pregnant patients (pH 7.38 (7.32–7.44)); HCO_3_^−^ 16.8 (16.2–22.2) mmol/L) were not different compared to nonpregnant controls (pH 7.45 (7.4–7.49); HCO_3_^−^ of 24 (22.8–25) mmol/L; *p* = 0.16 and 0.07, respectively) (Table 3). However, partial carbon dioxide pressure (paCO_2_) at admission was lower in the pregnant women (3.4 (3.1–3.7) kPa versus 4.5 (3.9–5.9) kPa; *p* = 0.007). The minimal recorded pH values during the first 7 days were equal in the pregnant group and in the nonpregnant group. The lowest HCO_3_^−^ was not different in the two groups (pregnant: 14.8 (12.8–18.6) mmol/L vs. nonpregnant: 22.7 (14.3–24.9) mmol/L (*p* = 0.097)). At this timepoint, paCO_2_ was also not different (pregnant 3.4 (3.3–4.5) kPa vs. non- pregnant 5.2 (4.2–5.9) kPa, *p* = 0.097). Six out of seven pregnant women suffered from a normal-AG metabolic acidosis; one of these cases was hyperchloremic acidosis, and two were mixed normal-AG and high-AG metabolic acidosis. Among the nonpregnant patients, two had hyperchloremic normal-AG metabolic acidosis (Supplementary File S1).

The urine analysis (Table 4) showed no difference in pH levels in pregnant and in non-pregnant patients (5.5 (5–6.5) vs. 6 (5.6–6.0); *p* = 0.73). The urine anion gap (UAG) in pregnant women was 33 (26.8–43.5) meq/L in a normal range. In the control group, the parameters to calculate UAG were only measured in one patient (39 meq/L).

Three out of seven pregnant COVID-19 patients (43%) fulfilled the diagnostic criteria of RTA [15], whereas none of the nonpregnant control patients (0%) fulfilled criteria for RTA. Given that the main differential diagnosis of hyperchloremic (non-anion gap) metabolic acidosis in patients with normal GFR is intestinal bicarbonate loss, we also reviewed charts for the presence of diarrhea but did not find it in any of the patients (Supplementary File S1). In three pregnant women, bicarbonate (NaHCO_3_^−^) was substituted over 2 (2–3) days. One patient of the control group also received NaHCO_3_^−^.

## 4. Discussion

Analyzing the acid–base characteristics of pregnant critically ill COVID-9 patients, it seems that metabolic acidosis is very common (85%) in these patients. Furthermore, in half of the women, history and biochemical parameters were suggestive of RTA. None of them showed impaired renal function regarding urine production or excretory function in the context of physiological pregnancy adaptations. Among the control group of COVID-19 women in the childbearing age, none had any metabolic acidosis and, consequently, none fulfilled the criteria for RTA. This was despite the fact that the patients were similarly ill according to the SOFA score on ICU admission and the SAPS score after 24 h. The ICU admission process is generally driven by multiple factors and, to a certain extent, dependent on the individual assessment of the attending physician. The simple presence of “pregnancy” in any patient that needs ICU evaluation might play a major additional psychological role in this complex decision-making process. One can speculate that this phenomenon is responsible for the trend that we observed in severity of disease between pregnant and nonpregnant COVID-19 patients. As discussed above, pregnant women are more vulnerable to disturbances in acid–base homeostasis, especially metabolic acidosis, mainly due to a reduced buffer capacity [6]. Therefore, reduced capacity of acid excretion easily becomes more relevant during pregnancy. Nevertheless, this concept cannot explain the high rate of RTAs that we observed in pregnant COVID-19 patients.

Within the last months, reports demonstrating that SARS-CoV-2 can infect literally any organ (including the kidney) via its tissue–blood barrier, i.e., the endothelium, have accumulated [16]. From the pathophysiological point of view, it remains to be demonstrated if our observation is indeed a direct viral effect on the tubular epithelium [17] or an indirect epiphenomenon via inflammatory processes. An immune-mediated process triggered by the SARS-CoV-2 infection similar to the mechanism proposed in patients with autoimmune disease, with immune-mediated damage of the acid secreting cells [3], is conceivable.

We believe that it is of importance to state that the diagnosis of RTA was made in only one of our seven patients during their ICU stay. The remaining diagnoses were established in our subsequent systematic analysis. This underlines the relevance of our findings with regard to the general awareness among intensivists treating critically ill pregnant COVID-19 patients. These findings are of importance because metabolic acidosis in pregnancy is associated with higher maternal and fetal risk [6] due to fetal hypoxemia as a consequence of reduced blood flow to the uterus. Furthermore, the fetus without functioning lungs and kidneys is physiologically incapable of compensating for a maternal acid–base derangement [6]. Maternal metabolic acidosis may impair fetal bone growth and development [2]. Timely correction of acidosis before irreversible damage occurs is crucial. That being said, the fact that many pregnant women are hesitant with regard to vaccination due to their unborn child has led to an increase in severe COVID-19 cases compared to nonpregnant women of the same age group (own unpublished data).

Interestingly, we saw a spontaneous recovery of metabolic acidosis in all women within 7 days during the ICU stay. Nevertheless, three of seven patients required bicarbonate substitution to correct the metabolic acidosis.

Our study had limitations. First, its retrospective and descriptive nature limited its general value compared to a controlled approach. In the future, we will follow up on this observation in a prospective design (in both pregnant COVID-19 and nonpregnant COVID-19 patients). Secondly, despite a cohort of more than 300 critically ill COVID-19 patients, only eight were pregnant. Even though the prevalence of RTA was very high in this cohort, the general sample size is rather low, thus limiting generalizability and highlighting the hypothesis-generating nature of our study.

## 5. Conclusions

Normal-AG metabolic acidosis seems to be very common (85%) in critically ill pregnant COVID-19 patients, and the prevalence of RTA might be higher than normal. It remains to be demonstrated if this observation is an indirect epiphenomenon or a direct viral effect on the tubular epithelium. However, in light of the known negative effects of acidosis for the unborn, we want to raise awareness of this seemingly common occurrence of an otherwise extremely rare disease in critically ill pregnant COVID-19 patients.

## Figures and Tables

**Table 1:** Treatment strategies according to group.

	Pregnant (*n* = 7)	Nonpregnant (*n* = 7)
HFOT (*n*, %)	5 (71)	3 (43)
Invasive MV (*n*, %)	3 (43)	5 (71)
ECMO (*n*, %)	0	3 (43)
Steroids (*n*, %)	6 (86)	6 (86)
Remdesivir (*n*, %)	0	3 (43)
Tocilizumab (*n*, %)	0	2 (29)
Convalescent plasma (*n*, %)	0	1 (14)

Abbreviations: HFOT (high-flow oxygen therapy), *n* (number) NIV (noninvasive ventilation), MV (mechanical ventilation), and ECMO (extracorporeal membrane oxygenation).

**Table 2:** Baseline characteristics.

	Pregnant (*n* = 7)	Nonpregnant (*n* = 7)	*p*-Value
Age (years)	31 (26–35)	29 (25–43)	1.0
BMI (kg/m^2^)	35 (26–43)	29 (22–33)	0.259
SOFA score admission	3 (3–6)	2 (2–6)	0.535
SOFA score maximal	6 (3–8)	8 (6–10)	0.209
SAPS (24 h)	19 (6–23)	22 (21–42)	0.053
LDH (U/L)	320 (284–553)	403 (313–453)	0.836
Ferritin (µg/L)	14 (40–285)	518 (443–766)	0.024
CRP (mg/L)	81 (56–131)	166 (42–317)	0.318
PCT(µg/L)	0.22 (0.13–0.26)	0.22 (0.09–8.8)	0.841
Lymphocytes (G/L)	0.76 (0.59–1.1)	0.81 (0.56–1.41)	0.62
D-dimers (µg/L)	2030 (720–2650)	980 (410–1560)	0.25
Creatinine (µmol/L)	42 (28–51)	48 (44–75)	0.073
eGFR (mL/min)	135 (123–158)	117 (100–131)	0.138
BUN (mmol/L)	1.1 (0.8–1.2)	3.1 (2.6–6.2)	0.002
Potassium (mmol/L)	3.1 (2.8–3.7)	3.9 (3.4–4.2)	0.053
Hospital stay (days)	12 (10–20)	28 (12–40)	0.209
ICU stay (days)	5 (5–8)	15 (10–36)	0.073

Abbreviations: ICU (intensive care unit), SOFA (Sequential Organ Failure Assessment), SAPS (Simplified Acute Physiology Score), LDH (lactate dehydrogenase), CRP (C-reactive protein), PCT (procalcitonin), IL-6 (interleukin-6), eGFR (estimated glomerular filtration rate, CKD-EPI 2009), and BUN (blood urea nitrogen).

**Table 3:** Blood gas analyses at ICU admission and lowest values during the first 7 days.

	Pregnant (*n* = 7)	Nonpregnant (*n* = 7)	*p*-Value
pH admission	7.38 (7.32–7.44)	7.45 (7.4–7.49)	0.16
pH min	7.3 (7.18–7.31)	7.31 (7.28–7.34)	0.535
HCO_3_^−^ admission (mmol/L)	16.8 (16.2–22.2)	24 (22.8–25)	0.07
HCO_3_^−^ min (mmol/L)	14.8 (12.8–18.6)	22.7 (14.3–24.9)	0.097
paCO_2_ admission (kPa)	3.4 (3.1–3.7)	4.5 (3.9–5.9)	0.007
paCO_2_ at minimal HCO_3_^−^ (kPa)	3.4 (3.3–4.5)	5.2 (4.2–5.9)	0.097
Cl^−^ (mmol/L)	112 (112–117)	112 (111–114)	0.383
AG	12 (8.2–16.1)	7.5 (4–9.3)	0.097

Abbreviations: HCO_3_^−^ (hydrogen carbonate), paCO_2_ (partial arterial carbon dioxide pressure), paO_2_ (partial arterial oxygen pressure), Cl^−^ (chloride), and AG (anion gap).

**Table 4:** Urine analysis.

Parameter	Pregnant (*n* = 7)	Nonpregnant (*n* = 7)	*p*-Value
Urine pH	5.5 (5–6.5)	6 (5.6–6.0)	0.73
Urine pH ≤ 5.3 (*n*)	2	0	
UAG	33 (26.8–43.5)	39 *	1.00

Abbreviations: UAG (urine anion gap). * Only one value.

## Data Availability

All data analyzed and discussed in the framework of this study are included in this published article and its online Supplementary Materials. The corresponding author may provide specified analyses or fully deidentified parts of the dataset upon reasonable request.

## Supplementary Materials

The following supporting information can be downloaded at https://www.mdpi.com/article/10.3390/jcm11154273/s1, Supplementary File S1: Individual acid base related characteristics.

## References

[1] Mir T., Almas T., Kaur J., Faisaluddin M., Song D., Ullah W., Mamtani S., Rauf H., Yadav S., Latchana S. (2021). Coronavirus disease 2019 (COVID-19): Multisystem review of pathophysiology. Ann. Med. Surg..

[2] Varga Z., Flammer A.J., Steiger P., Haberecker M., Andermatt R., Zinkernagel A.S., Mehra M.R., Schuepbach R.A., Ruschitzka F., Moch H. (2020). Endothelial cell infection and endotheliitis in COVID-19. Lancet.

[3] Farkash E.A., Wilson A.M., Jentzen J.M. (2020). Ultrastructural evidence for direct renal infection with SARS-CoV-2. J. Am. Soc. Nephrol..

[4] Fujii T., Udy A.A., Nichol A., Bellomo R., Deane A.M., El-Khawas K., Thummaporn N., Serpa Neto A., Bergin H., Short-Burchell R. (2021). Incidence and management of metabolic acidosis with sodium bicarbonate in the ICU: An international observational study. Crit. Care.

[5] Gunnerson K.J. (2005). Clinical review: The meaning of acid-base abnormalities in the intensive care unit part I—Epidemiology. Crit. Care.

[6] Morris R.C. (1969). Renal tubular acidosis. Mechanisms, classification and implications. N. Engl. J. Med..

[7] Vallés P.G., Batlle D. (2018). Hypokalemic distal renal tubular acidosis. Adv. Chronic Kidney Dis..

[8] Palmer B.F., Kelepouris E., Clegg D.J. (2021). Renal tubular acidosis and management strategies: A narrative review. Adv. Ther..

[9] Williams D., Davison J. (2008). Chronic kidney disease in pregnancy. BMJ.

[10] Harel Z., McArthur E., Hladunewich M., Dirk J.S., Wald R., Garg A.X., Ray J.G. (2019). Serum creatinine levels before, during, and after pregnancy. JAMA.

[11] Seeger H. (2018). Säure-Basen-Störungen in der Schwangerschaft. Der. Nephrol..

[12] Seeger H., Salfeld P., Eisel R., Wagner C.A., Mohebbi N. (2017). Complicated pregnancies in inherited distal renal tubular acidosis: Importance of acid-base balance. J. Nephrol..

[13] Firmin C.J., Kruger T.F., Davids R. (2007). Proximal renal tubular acidosis in pregnancy. A case report and literature review. Gynecol. Obstet. Investig..

[14] Narcisse D., Agarwal M., Kumar A. (2017). A rare case of transient proximal renal tubular acidosis in pregnancy. Case Rep. Nephrol..

[15] Yaxley J., Pirrone C. (2016). Review of the diagnostic evaluation of renal tubular acidosis. Ochsner J..

[16] Jonigk D., Märkl B., Helms J. (2021). COVID-19: What the clinician should know about post-mortem findings. Intensive Care Med..

[17] Puelles V.G., Lütgehetmann M., Lindenmeyer M.T., Sperhake J.P., Wong M.N., Allweiss L., Chilla S., Heinemann A., Wanner N., Liu S. (2020). Multiorgan and Renal Tropism of SARS-CoV-2. N. Engl. J. Med..

