# Supplementary material for: Renal Tubular Acidosis in Pregnant Critically Ill COVID-19 Patients: A Secondary Analysis of a Prospective Cohort
Source: J Clin Med. 2022 Jul 22;11(15):4273. doi: 10.3390/jcm11154273 (PMC9331067; doi:10.3390/jcm11154273)
Supplement: Supplementary file 1 [file jcm-11-04273-s001.zip › jcm-1775112-supplementary.pdf]

Supplementary File S1

Supplementary File S1: Individual acid base related characteristics

Patient	Pregnant	Metabolic Acidosis	AG calculated	Cl^-	Diarrhea	dRTA	Interpretation
1	Yes	Yes	8.2	116	No	Yes	
2	Yes	Yes	9.2	117	No	Yes	
3	Yes	Yes	14.3	112	No	No	Hyperchloremic normal anion gap acidosis
4	Yes	Yes	17.5	124	No	No	High anion gap acidosis
5	Yes	Yes	6.5	112	No	Yes	
6	Yes	No	12	110	No	No	
7	Yes	Yes	16.1	112	No	No	High anion gap acidosis
**Ø**	**7/7**	**6/7**	**12.0**	**115**	**0/7**	**3/7**	
1	No	No	9.1	111	No	No	
2	No	Yes	7.5	114	No	No	Hyperchloremic normal anion gap metabolic acidosis
3	No	No	4	112	No	No	
4	No	No	9.3	112	No	No	
5	No	No	3.1	111	No	No	
6	No	Yes	14.4	123	No	No	Hyperchloremic normal anion gap metabolic acidosis
7	No	No	6.5	108	No	No	
**Ø**	**0/7**	**2/7**	**7.7**	**113**	**0/7**	**0/7**	
